# Knowledge, attitudes and practices to hepatitis B among South African primary healthcare staff

**DOI:** 10.4102/phcfm.v17i1.4646

**Published:** 2025-01-22

**Authors:** Atlegang Mashilo, Itumeleng Mompati, Refilwe Ramakatane, Didintle Sebitloane, Naledi Sibi, Philippa C. Matthews, Cornel Van Rooyen, Dominique Goedhals, Sabeehah Vawda

**Affiliations:** 1 Department of Clinical Medicine, Faculty of Health Sciences, University of the Free State, Bloemfontein, South Africa; 2 The Francis Crick Institute, London, United Kingdom; 3 Department of Infection and Immunity, University College London, London, United Kingdom; 4 Department of Infectious Diseases, University College London Hospital, London, United Kingdom; 5 Department of Biostatistics, Faculty of Health Sciences, University of the Free State, Bloemfontein, South Africa; 6 Department of Virology, Faculty of Health Sciences, University of the Free State, Bloemfontein, South Africa; 7 National Health Laboratory Service, Bloemfontein, South Africa; 8 Department of Virology, Pathcare Laboratory, Pretoria, South Africa

**Keywords:** hepatitis B virus, primary healthcare, hepatitis, knowledge, health education, awareness, prevention

## Abstract

**Background:**

Hepatitis B virus (HBV), a significant cause of liver disease globally, is recognised as a 2030 elimination target by the World Health Organization (WHO). Healthcare workers (HCWs) require appropriate HBV knowledge to identify, manage and prevent HBV.

**Aim:**

We investigated the knowledge, attitude and practices (KAP) pertaining to HBV among HCWs to establish insights into awareness and inform the delivery of training.

**Setting:**

The study was conducted among HCWs of 18 primary healthcare facilities in Bloemfontein, Free State province, South Africa.

**Methods:**

Data were collected via anonymous, self-applied, 28-question-questionnaires in English. Data were captured on a Microsoft Excel spreadsheet and analysed by a biostatistician, using Statistical Analyses Software (SAS 9.4).

**Results:**

The response rate was 88% (88/100), and median participant age was 44 years. Participants were mostly female (83%), professional nurses (65%) with more than 8 years of experience (60%). Median scores were 83% for epidemiology and transmission, 50% for clinical picture, 44% for laboratory diagnosis, 40% for management and 40% for prevention. No difference was noted based on number of years of experience.

**Conclusion:**

Considerable gaps in KAP to HBV were noted among primary HCWs in Bloemfontein. Larger studies are needed to ascertain the KAP towards HBV among South African HCWs, to identify areas for enhanced training.

**Contribution:**

Hepatitis B virus, an important cause of liver disease in Africa, is poorly identified and managed. Our study highlights the need to strengthen HCW education to ensure individuals are appropriately diagnosed, managed and educated on preventative measures, to reduce the burden of disease.

## Introduction

Hepatitis B virus (HBV) is a significant cause of liver disease worldwide, leading to the potentially fatal complications of cirrhosis and hepatocellular carcinoma (HCC). According to 2019 World Health Organization (WHO) estimates, 296 million people globally were chronically infected with HBV, with 7.5% of the general population in the WHO Africa region infected.^1,2^ Viral hepatitis was officially acknowledged as a global health priority in 2015 and was included as a focus area in the health-related goal of the 17 Sustainable Development Goals of the United Nations 2030 Agenda for Sustainable Development. The targets to be achieved by 2030 include 90% reduction in new cases of chronic HBV, 90% of HBV cases diagnosed, 80% of treatment-eligible persons on treatment, and 90% prevention of HBV mother-to-child transmission.^3^ In order to meet these targets, there is a pressing need to ensure that healthcare workers (HCWs) have appropriate Knowledge, Attitudes and Practices (KAP) such that they can protect themselves, and offer relevant information, education and health interventions within their communities.

Despite the availability of a safe, efficacious vaccine, the WHO estimates that in 2019 approximately 1.5 million people newly acquired HBV, with the WHO African region accounting for 66% (*n* = 990 000) of these.^1^ Estimates suggest that, only 2% (*n* = 1.8 million) of people living with HBV in the African region knew their status in 2019, with 0.1% (*n* = 110 000) accessing treatment.^1^ While the exact prevalence of chronic HBV in South Africa is unclear, available data suggest the prevalence is intermediate (2% – 7%) to high (≥ 8%).^4,5,6,7^

In an effort to reduce the development of chronic HBV, South Africa introduced the HBV vaccine as part of the South African Expanded Programme on Immunisation (EPI) in 1995, with doses at 6, 10 and 14 weeks of age, followed by a booster dose at 18 months of age. The introduction of an HBV birth dose vaccine remains in process. In addition, South African Guidelines recommend the HBV vaccine for all HCWs, student HCWs, staff in healthcare facilities at risk for exposure and laboratory workers handling patient samples. Post-immunisation antibody testing is recommended to ensure the correct follow-up and management of vaccine non-responders.^8^

A recent laboratory-based, country-wide study reported a consistent decrease in HBsAg (Hepatitis B surface antigen) test positivity and the incidence of acute HBV infection over a 5-year period, from 2015 to 2019, suggestive of the positive impact of the HBV vaccine into the EPI schedule.^9^ In a corresponding study, the researchers described a 4.83% HBsAg test positivity in the below 5-year age category, with active infections significantly higher in children less than 1 year old, highlighting the need for enhanced preventative methods including a birth dose of HBV and screening and management of pregnant women.^10^

Hepatitis B virus treatment with nucleos(t)ide analogue therapy is currently offered based on algorithms that aim to identify those at highest risk of complications, and is based on clinical evaluation, laboratory testing and imaging. However, guidelines are expected to change to simplify this assessment and to widen eligibility for treatment. A combination of maternal antiviral prophylaxis and vaccination can be used to prevent vertical transmission.^8^

In Africa, studies among HCWs have shown varying levels of knowledge. In a study conducted in Sierra Leone among 211 HCWs, 30% were uncertain about transmission routes, 77% were unaware of the clinical effects of HBV infection and 44% were not well informed of preventative measures.^11^ A KAP study conducted among 175 HCWs in Ghana, reflected similar knowledge gaps.^12^ In contrast, a study conducted in Ethiopia found that 73% of 297 HCWs had good knowledge of HBV transmission, progress and vaccination,^13^ while a Sudanese study found that 96% of respondents knew the appropriate method of diagnosing an individual with HBV.^14^

It is therefore evident that the KAP of HCWs on HBV differs both among and within different countries. The KAP towards HBV among South African HCWs is unknown, with no published studies to date. While HBV vaccination has been included in the South African EPI almost 30 years prior, recent data indicate that HBV remains relevant in our setting, especially at the primary healthcare (PHC) level. We therefore set out to evaluate the KAP pertaining to HBV among HCWs in the Free State province of South Africa to establish insights into awareness and to inform the delivery of information and education.

## Research methods and design

### Study design

A descriptive cross-sectional study was employed to determine the KAP pertaining to HBV infection among HCWs at PHC clinics in the Free State, South Africa.

### Study setting

**FIGURE 1:**
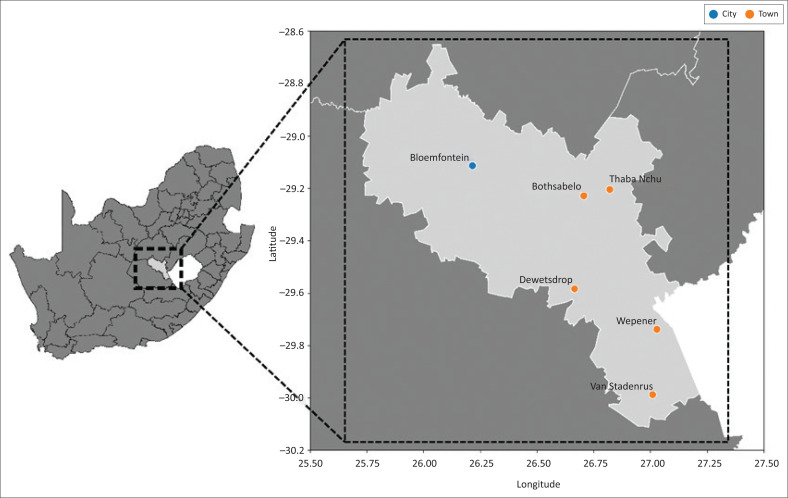
Map of the Mangaung Metropolitan Municipality, Free State, South Africa, depicting the city of Bloemfontein and other major towns.

Our study was conducted among primary HCWs at selected PHC facilities in Bloemfontein in 2020. Bloemfontein is a city forming part of the Mangaung metropolitan, located in the central, mainly rural, Free State province of South Africa. The Mangaung Metropolitan Municipality (Figure 1) covers a land size of 9886 km^2^ and has a population of 861 651, with Bloemfontein, one of three urban centres, being home to approximately 580 000 people.^15,16^

The healthcare system in South Africa is two-tiered, consisting of public and private health sectors. The state-funded public health sector, including laboratory services, is utilised by 71.9% of all South Africans and is divided into primary, secondary and tertiary services. The Mangaung metropolitan consists of four health sub-districts with a total of 313 clinics and 122 mobile clinics.^15,16,17^

The Bloemfontein sub-district is served by 20 clinics and 2 community health centres (CHCs).^18^ A total of 18 PHC facilities in the Bloemfontein sub-district, with the exclusion of mobile clinics, were selected. This included 2 CHCs and 16 fixed community clinics, selected because of ease of access via public transport for the researchers. Facility names are withheld to maintain anonymity. Community health centres are larger healthcare clinics that operate 24-h a day and always have a doctor on site. These manage a wide range of conditions including uncomplicated obstetric deliveries and eye and dental services. Community clinics are smaller clinics that operate during working hours from Monday to Friday. These are primarily managed by nursing staff with a doctor providing support for more complex cases on specific days.^19^

### Study population

We aimed to include all HCWs employed at the 18 PHC facilities in the Bloemfontein sub-district, subject to study inclusion and exclusion criteria. All HCWs employed at the various PHC facilities, including doctors and nurses, professional nurses, enrolled nurses, enrolled midwives and enrolled nursing assistants, who volunteered to participate in the study, were included. We excluded all HCWs not employed at the various PHC facilities. Healthcare workers and staff other than doctors and nurses namely, administrative staff, cleaning and maintenance staff and allied healthcare personnel were also excluded from our study cohort.

We distributed a total of 100 questionnaires to the 18 PHC facilities, based on our estimated study population of 100 HCWs. We contacted selected clinics to determine the numbers and categories of staff employed. Based on this interaction, we estimated the CHCs to have approximately 25–35 HCWs while the community clinics to have a minimum of 1–2 eligible HCWs. A minimum of 80 HCWs’ responses was considered a representative response.

### Sampling strategy

The researchers contacted the clinic manager or sister-in-charge at each of the 18 PHC facilities by telephone to inform them of the intended study as per participant study information document (Online Appendix 1: Data B). Permission was sought to visit the relevant PHC facilities to deliver the study information documents and questionnaires for voluntary staff participation.

The researchers travelled to the 18 PHC facilities and met with the clinical lead at each site to provide hard copies of the study information documents and questionnaires for distribution among the facility staff, as per study inclusion and exclusion criteria.

The questionnaires were collected after a period of 2 weeks, to allow staff working different shifts and those on leave, an opportunity to participate. Participants were asked to complete the questionnaire independently, without referring to any sources of information.

### Data collection

We collected data via the distribution of an anonymous self-completed questionnaire in English (Online Appendix 1: Data A). The constitution of South Africa recognises 11 official languages with English accepted as one of the official languages by the National Department of Health (NDOH).^20^

The questionnaire included demographic details such as age, gender, occupation and years of experience, followed by 28 knowledge and practice-based questions and statements to assess the KAP of HCWs regarding the epidemiology (*n* = 7), clinical picture (*n* = 2), laboratory diagnosis (*n* = 9), management (*n* = 5) and prevention (*n* = 5) of HBV. Participants had three options as a response to each question or statement, ‘Yes’, ‘No’ or ‘Unsure’. This close-ended question option was used to ensure standardisation in the answers. Study participants received a score of 1 for each correct answer and a score of 0 for incorrect and ‘Unsure’ answers.

### Pilot study

We conducted a pilot study in August 2020 to test the use of the planned questionnaire, by distributing questionnaires to one PHC clinic. Five participants meeting the eligibility criteria, completed the questionnaire and were asked for feedback. There were no concerns regarding understandability or ambiguity. The questionnaire was therefore taken forward without any amendments. The data obtained from the pilot study were included in the results of the main study.

### Data analysis

Data from the completed questionnaires were captured on a Microsoft Excel spreadsheet. All Excel sheets were password protected with access limited to the researchers only. Numerical variables were summarised by medians, minimum, maximum or percentiles. Categorical variables were summarised by frequencies and percentages. Differences between groups and numerical variables were evaluated using the Wilcoxon Two-Sample test for unpaired data. The analysis was done by the Department of Biostatistics, using Statistical Analysis Software (SAS 9.4, Statistical Analysis Software Institute Inc., Cary, NC, US).

### Ethical considerations

Questionnaires were anonymous with no identifying information collected, and participation was completely voluntary. Approval to conduct the study was obtained from the Health Sciences Research Ethics Committee (HSREC) of the University of the Free State (UFS-HSD2020/0610/2508) as well as from the Free State Department of Health (FSDoH).

## Results

### Characteristics of study participants

We distributed 100 questionnaires to 18 PHC facilities in Bloemfontein, South Africa, based on our estimated study population of 100 HCWs. A total of 88 HCWs completed the questionnaires resulting in a response rate of 88% (88/100). The age of the participants ranged from 21 to 62 years (median 44 years, 86/88 provided a response). Participants were predominantly female (73/88, 83%), professional nurses (57/88, 65%) and had more than 8 years of experience (53/88, 60%), (Table 1). The occupation field was marked as other (23/88, 26%) or left unanswered (5/88, 6%) in 32% of questionnaires. These participants could be enrolled nurses, enrolled midwives or enrolled nursing assistants who work at the PHC facilities but have a different scope of practice from professional nurses.

**TABLE 1:** Demographic characteristics of the study participants at primary healthcare clinics in Bloemfontein, South Africa (*N* = 88).

Variables	Number	%
**Gender**		
Female	73	83
Male	15	17
**Occupation**		
Professional nurse	57	65
Medical doctor	3	3
Other	23	26
Unanswered	5	6
**Years of experience**		
< 1	8	9
1–4	13	15
4–8	10	11
> 8	53	60
Unanswered	4	5

### Knowledge, attitudes and practices of healthcare workers on hepatitis B virus epidemiology and transmission routes

**FIGURE 2:**
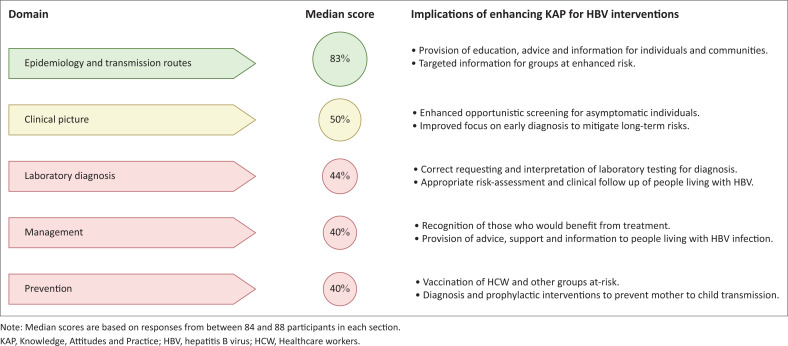
Median score of Knowledge, Attitudes and Practice pertaining to hepatitis B virus infection among healthcare workers at primary healthcare clinics in the Free State, South Africa, and suggested interventions.

All 88 participants completed this section consisting of seven questions. Details regarding the number answered correctly, incorrectly and unanswered are provided in the Online Appendix 1: (Table S1). Participants scored highest in this section with a median score of 83% (Interquartile range [IQR] 28.6%–100.0%) (Figure 2). A total of 68 (77%) participants scored ≥ 70% with eight (9%) participants scoring ≤ 50%.

Closer analysis of individual questions showed that only 62% (54/87) knew that HBV is common in South Africa and only 42% (37/88) knew that HBV transmission can occur horizontally through household and/or close contact. A total of 66/86 (77%) and 65/87 (75%) knew that HBV can be sexually transmitted and perinatally transmitted, respectively.

### Knowledge, attitudes and practices of healthcare workers on the clinical picture of hepatitis B virus

The median score for this section consisting of two questions was 50% (Figure 2). Twenty-one (24%) participants scored 0, 46 (54%) scored 50% and 18 (21%) scored 100% for this section. Only 24% (20/83) of the participants knew that individuals with hepatitis B infection are often asymptomatic (Online Appendix 1: Table S2).

### Knowledge, attitudes and practices of healthcare workers on the laboratory diagnosis of hepatitis B virus

Participants exhibited limited knowledge of the laboratory diagnosis of HBV with a median score of 44% (IQR 0%–100%) (Figure 2) for the nine questions in the section (Online Appendix 1: Table S3). Forty-eight (55%) participants scored ≤ 50%.

Only 10% (8/83) of the participants knew that infection with HBV cannot be clinically differentiated from other causes of hepatitis. Sixty-four per cent (51/80) of the participants knew that the hepatitis B surface antigen test is used to diagnose infection, and 58% (48/83) were aware that hepatitis B surface antibody is a marker of HBV immunity. Most participants (90%, 74/82) were aware that people with HIV should be tested for HBV co-infection. More than half of the participants were unaware or unsure (55%, 47/85) about the availability of hepatitis B viral load testing.

### Knowledge, attitudes and practices of healthcare workers on the management of hepatitis B virus

Participant knowledge regarding the management of HBV was low, with participants scoring a median of 40% (IQR 0%–100%) (Figure 2) for the five questions. Forty-six (52%) participants scored ≤ 50%.

Many participants incorrectly stated that acute HBV required antiviral treatment (58%, 49/84) or were unsure (29%, 24/84) (Online Appendix 1: Table S4). Just over half of the participants (55%, 46/84) were aware that chronic HBV is treatable with Tenofovir, with 35% (29/84) of the participants stating uncertainty. Most participants (76%, 64/84) incorrectly stated that HBV is curable.

### Knowledge, attitudes and practices of healthcare workers on prevention of hepatitis B virus

Regarding the prevention of HBV, participants scored a median of 40% (IQR 0%–100%) (Figure 2) for the five questions. A total of 46 (52%) participants scored ≤ 50%.

Most participants were aware that HBV is vaccine preventable (93%, 77/83) and that all HCWs should be vaccinated against HBV (96%, 79/82), but there was uncertainty regarding the antibody titer deemed to be protective, with only 63% (51/81) answering correctly (Online Appendix 1: Table S5). Most participants (88%, 74/84) knew that HBV vaccine forms part of the South African EPI. Only 64% (53/83) of the participants knew that babies born to mothers with HBV require both a birth dose vaccine and may be eligible for hepatitis B immunoglobulin.

### Knowledge, attitudes and practices of healthcare workers on hepatitis B virus according to years of experience

No difference was noted in the median knowledge score of participants based on the number of years of experience (Table 2).

**TABLE 2:** Median knowledge score for the different questionnaire sections according to years of experience.

Questionnaire section	Variables				*p*
Years of experience	< 1	1–4	4–8	≥ 8	-
Epidemiology and transmission routes (%)	83	71	86	86	0.24
Clinical picture (%)	50	50	50	50	0.81
Laboratory diagnosis of HBV (%)	50	44	47	47	0.26
Management of HBV (%)	60	40	40	40	0.65
Prevention of HBV (%)	60	40	40	40	0.65

HBV, hepatitis B virus.

## Discussion

Our study conducted in the Free State province, is the first study investigating KAP among primary HCWs regarding HBV in South Africa. Participants had the highest median score for HBV epidemiology but the scores for HBV clinical picture, laboratory diagnosis, management and prevention were lower. The number of years of experience did not impact on knowledge scores. This suggests that HCWs do not gain knowledge through training or experience delivered within the workplace, and need more information and education, irrespective of their career stage. Reasons for gaps in HBV knowledge were not specifically explored by this study but may include a lack of inclusion in training curricula, personal reasons, a lack of time, and a lack of provision of educational opportunities by the provincial department of health.

The profile of predominantly female primary HCWs corresponds to the usual demographics of PHC staff in South Africa. According to the 2022 South African Nursing Council statistics, the female: male ratio of nurses in South Africa was 8:1 (*n* = 241 950 females, *n* = 29 097 males) with a ratio of 6:1 (*n* = 10 881 females, *n* = 1911 males) for the Free State province.^21^

Participants had the highest median knowledge score for HBV epidemiology; however, an under-recognition of the prevalence means that HCWs may not consider providing education, diagnostic testing or preventive information, including appropriate advice on precautions to prevent transmission (e.g. use of condoms, partner and/or household vaccination, antiviral treatment, interventions to prevent mother-to-child transmission). A minority of HCWs were aware of the potential for mother-to-child transmission of HBV, which suggests that crucial opportunities are missed for education, testing, providing treatment or prophylaxis and/or referral to specialist antenatal services. While the risk of acquiring HBV from needle stick injuries was common knowledge, it was less well recognised that HBV-infected HCWs could transmit the virus to their patients. This implies a lack of integration of transmission knowledge into clinical practice.

Lack of recognition that HBV is often asymptomatic suggests that screening opportunities are likely to be missed. A third of HCWs were unaware of the risk for HCC, highlighting the lack of insights into morbidity and mortality associated with chronic HBV infection.

Our results indicate considerable uncertainty regarding interpretation of routine serological testing. This implies that while HCWs may follow guidelines in requesting laboratory tests, the results may not be acted on appropriately. This underlines the importance of having laboratory interpretive comments on result reports, and suggests a role for reflex testing, such that laboratory protocols provide and report a relevant serological profile. Hepatitis B viral load testing is available in South Africa but is currently underutilised, with >50% of HCWs being unaware or uncertain about this laboratory test.

There was good awareness that patients with HIV should be tested for HBV co-infection, and majority of the participants were aware of the necessity of including Tenofovir in the antiretroviral regimens of patients co-infected with HIV and HBV. Together, these results indicate good awareness of the current HIV clinical guidelines in South Africa, which provides a good foundation for enhanced provision of training in HBV.^22^

Our study population was generally not confident with regards to the correct management of patients diagnosed with HBV, with >75% believing HBV infection to be curable, showing a lack of knowledge of the natural history of HBV.

Primary HCWs are the first and often the only immediate access to healthcare services for the majority of the South African population. Understanding the basics of HBV is critical for primary HCWs in order to correctly diagnose, manage and prevent HBV.

### Limitations

Our study population size and distribution of questionnaires were based on a calculation of the estimated number of HCWs at the PHC clinics. Our study population therefore does not necessarily represent the actual number of HCWs and may have under- or over-estimated the number of HCWs. The results may therefore be biased and not accurately represent the KAP of hepatitis B among all HCWs in Bloemfontein.

Our sample size was small, and we cannot exclude a possible bias as staff who elected to participate may be those with special interests or experience in managing HBV. On these grounds, our data may over-estimate knowledge. Conversely, our data may under-estimate knowledge if more inexperienced staff participated.

Participation in this study may also have been influenced by competing priorities during the coronavirus disease 2019 (COVID-19) pandemic. Because the questionnaires were left to be distributed by the facility manager, it is unknown whether staff from all work shifts were invited to participate. In addition, because questionnaire completion was not monitored, it is unknown if participants utilised resources for information.

The questionnaire was prepared only in the English language. While English is commonly used in the healthcare setting, this may have potentially hindered participation. Some participants left certain questions unanswered despite having the option of ‘Unsure’ on the questionnaire; the possibility of not understanding the question cannot be ruled out.

The questionnaire was designed with a free text space next to the option ‘Other’ for profession. Unfortunately, all participants who selected ‘Other’ did not specify their profession. Because the questionnaire entry was not monitored by the researchers, this information was not acquirable; therefore, we were unable to analyse the KAP according to profession or professional level.

The questionnaire was distributed as a paper copy only. Online availability may have increased participation; however, we decided against this because of potential participant issues with access and technical difficulties.

### Recommendations

Provincial departments of health should steer the process of ensuring HCWs at primary health level are included in HBV training events and keep abreast of new developments, particularly as the national and global landscape of treatment is likely to change, and care will need to be decentralised in order to reach more people with diagnostic testing and treatment. Hepatitis B virus could be included in future Continuing Professional Development (CPD) programmes in face-to-face or online modules. The South African Nursing Council has commenced the process of developing a mandatory CPD programme for South African nurses and midwives and our HBV study results provide a single example of the need to expedite this process.^23^ Primary healthcare facilities should ensure that time is dedicated to teaching, training and discussing current guidelines.

Hepatitis B virus should not be managed in a silo, but through programmes that combine opportunities for intervention in a variety of domains (e.g. combining with maternal health and triple elimination of HBV/HIV/syphilis, offering as part of family planning, sexual health, vaccine campaigns covering diverse infections and other community health initiatives).

Free text feedback could be helpful in future studies to add a qualitative component and to enhance insights into the reasons for limited knowledge and to harness suggestions from HCWs for building better awareness.

## Conclusion

Our study identified considerable gaps in KAP among primary HCWs in Bloemfontein, Free State, regarding HBV. Further studies involving a larger number of participants from different provinces, consisting of areas of different population densities as well as HCWs at different levels of healthcare are needed to build a more robust picture and identify priorities for training and teaching. This can inform decisions on more targeted outreach programmes to enhance knowledge and practices of diagnosing, managing and preventing HBV in order to reach the WHO and UN target goals to eliminate HBV.
